# Rectal gastrointestinal stromal tumor with early liver metastasis misdiagnosed as vaginal wall tumor: a rare case report

**DOI:** 10.3389/fonc.2025.1571654

**Published:** 2025-09-08

**Authors:** Xiuzhang Yu, Zhijiang Fang, Liyan Huang, Mingrong Qie, Xiumei Xu, Bowen Yang, Ruiqi Duan

**Affiliations:** ^1^ Department of Obstetrics and Gynecology, West China Second University Hospital, Sichuan University, Chengdu, China; ^2^ Key Laboratory of Birth Defects and Related Diseases of Women and Children (Sichuan University), Ministry of Education, Chengdu, China; ^3^ Department of Obstetrics and Gynecology, ChengDu PiDu District People’s Hospital, Chengdu, China; ^4^ Department of Pathology, West China Second University Hospital, Sichuan University, Chengdu, China

**Keywords:** rectal gastrointestinal stromal tumor, vaginal tumor, liver metastasis, imatinib, case report

## Abstract

Rectal gastrointestinal stromal tumors (GISTs) are rare mesenchymal tumors of the gastrointestinal tract. They lack specific clinical manifestations and imaging characteristics, leading to a high risk of misdiagnosis and missed diagnosis. Here, we describe a 49-year-old female initially diagnosed with a vaginal wall tumor who underwent vaginal tumor resection surgery. The final diagnosis was a high-risk gastrointestinal stromal tumor (epithelioid type). Uniquely, one month after surgery, enhanced CT and PET/MR/CT scans indicated liver metastasis. The patient underwent a partial resection of the left liver and was subsequently treated with oral imatinib. Six months post-surgery, the patient has shown no signs of recurrence. This case accentuates the need for clinicians to improve their understanding of this disease to reduce the rates of misdiagnosis and missed diagnosis. Surgical resection is an effective treatment for localized rectal GISTs, but vigilance for distant metastasis is essential. Enhanced CT and PET/MR/CT scans are necessary. Female patients with GISTs located on the anterior rectal wall may be suitable for a vaginal approach surgery, however, the indications need further study. The importance of early differential diagnosis of rectal GISTs need to be highlighting.

## Introduction

Rectal gastrointestinal stromal tumors (GISTs) are relatively rare, with an incidence of 0.018 per 100,000 person-years, accounting for approximately 0.1% of all rectal tumors and 2.8% of all GISTs (1). The clinical manifestations of rectal GISTs are typically nonspecific, and imaging often lacks specificity, early detection of the tumor is challenging, determine the tumor’s origin is also difficult. Clinicians, especially gynecologists, often lack awareness and vigilance regarding this disease, leading to a high risk of misdiagnosis. In this report, we present a case of rectal GIST misdiagnosed as a vaginal wall tumor, which led to a “vaginal tumor resection.” Uniquely, the patient developed liver metastasis just one month post-surgery without local recurrence. This case report highlights the diagnostic and therapeutic challenges of rectal GISTs, emphasizing the need for heightened awareness to prevent misdiagnosis and missed diagnosis.

## Case presentation

A 49-year-old female patient was admitted to the gynecology department following the discovery of a pelvic mass during a routine physical examination. The patient reported no symptoms such as abdominal pain, bloating, abnormal vaginal bleeding, or changes in bowel habits. On specialist examination, the vulva appeared normally, the vagina was patent. A palpable mass approximately 5 cm in size was detected on the lower vaginal wall, which was movable. The vaginal mucosa was normal, there was no bleeding from the cervical canal, and no obvious abnormalities were detected in the uterus or bilateral adnexa. The patient had no comorbidities. Ultrasound indicated a mixed cystic-solid mass measuring approximately 5.9 x 3.2 cm with an irregular shape and poorly defined boundaries located at the posterior vagina. The mass had an indistinct demarcation from the vaginal wall, and blood flow signals were observed within and around the mass.

**Figure 1:**
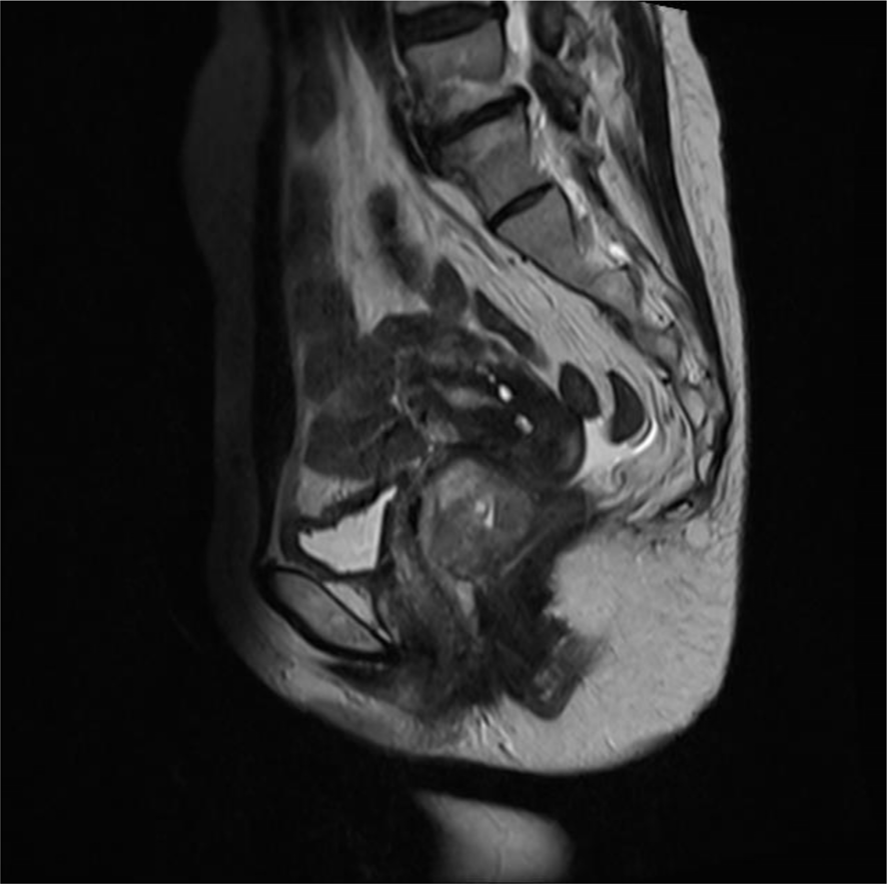
MRI shows a mixed, slightly prolonged T1 and T2 signal mass in the upper segment of the vagina.

Pelvic MRI revealed a mixed slightly hyperintense T1 and T2 signal mass in the upper vagina (**Figure 1**). The mass exhibited restricted diffusion on DWI, had an irregular shape, and measured approximately 5.9 x 3.9 x 3.6 cm. The posterior vaginal wall was discontinuous, and the mass extended posteriorly, obliterating the adjacent rectal space. The bilateral levator ani muscles remained continuous, though the right side was slightly compressed. The anterior wall was continuous, but the mass had an unclear demarcation from the external cervical os. There was no thickening of the bladder or rectal walls. Multiple small lymph nodes were visible in the bilateral obturator and inguinal regions. CA19–9 was 37.51 U/ml, and other tumor markers were within normal limits.

Considering the possibility of a vaginal wall tumor, a surgical plan was made to perform a transvaginal excision of the mass. Upon incision of the posterior vaginal wall, a 3x6 cm mass with a soft, irregular shape and friable tissue was discovered. The mass was densely adherent to the anterior rectal wall and infiltrated the rectal muscle layer. The mass was excised and sent for frozen section examination, which revealed a spindle cell tumor with some regions showing epithelial-like characteristics and atypia. The tumor exhibited pushing growth with indistinct boundaries from the surrounding tissue. The frozen section suggested at least a low-grade malignancy, pending further diagnosis with paraffin sections and immunohistochemical staining. After discussing the diagnosis and associated risks with the patient, we decided to proceed with lesion excision only. The procedure included repairing the anterior rectal wall and suturing the vaginal wall, with a rectal tube placed postoperatively.

**Figure 2:**
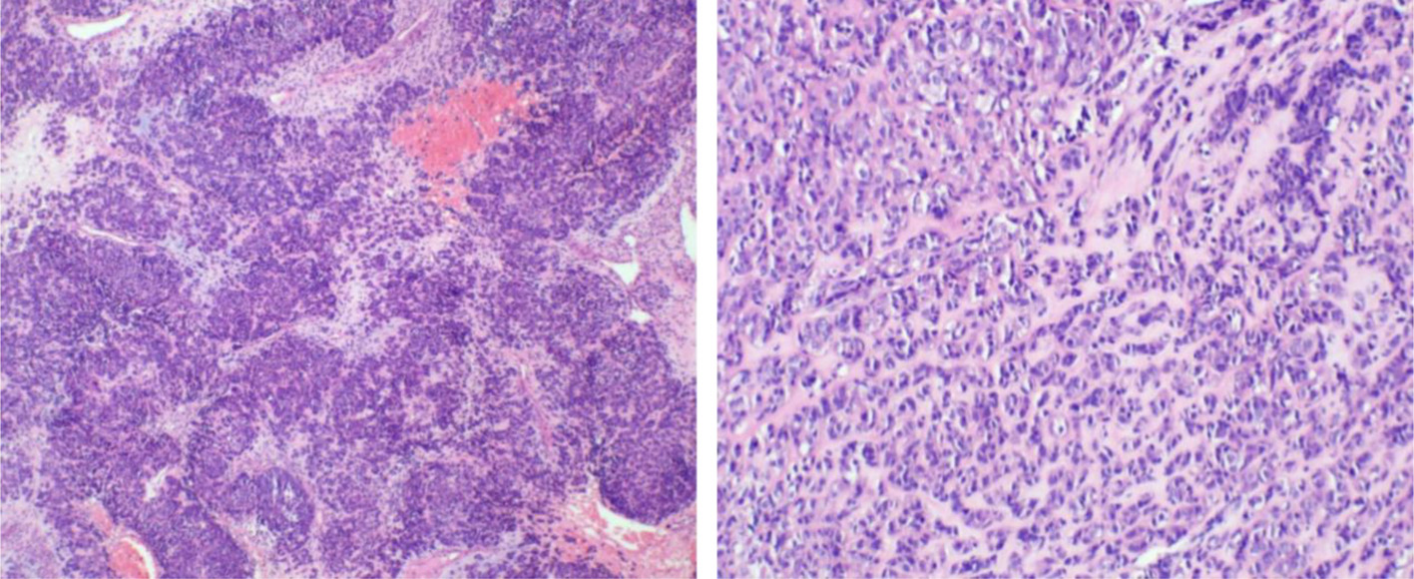
The tumor exhibits a sheet-like or nest-like arrangement, resembling epithelial cells with eosinophilic cytoplasm, which is partially clear or vacuolated, containing atypical cells with hyperchromatic nuclei. There is stromal hemorrhage and extensive collagenization.

**Figure 3:**
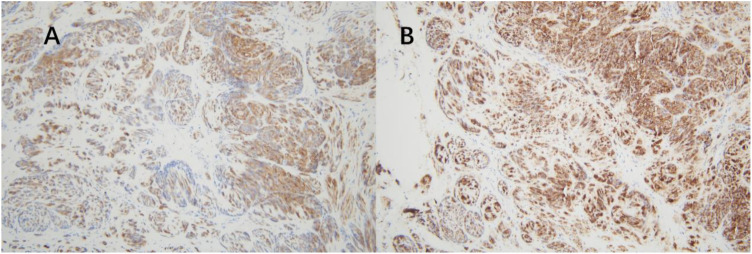
Immunohistochemistry. (A) positive for CD117. (B) positive for DOG-1.

Post-surgery, the tissues were sent to the pathology department at West China Second Hospital. Considering the characteristics of the tumor cells and limited immunohistochemical findings, a diagnosis of epithelioid hemangioendothelioma originating from vascular endothelium was considered (**Figure 2**). Immunohistochemistry (IHC) showed Vim multifocal positivity, CvclinD1 (++), AR (++), CD31 (++), CD34 (+++), EMA multifocal positivity, and nuclear expression of β-catenin (+++), CD117 multifocally positive, P53 showed wild-type expression, and P16, Ber-EP4, TIF-1, BCOR, E-Cad, Ck-P, Ck7, CA125, CD10, caldesmon, HER-2, and CAM5.2 were negative. Further examination at the pathology department of West China Hospital revealed negative results for CK, partial weak positivity for P63, and negativity for PAX8, ER, PR, GATA3, S-100, CgA, Syn, SMA, desmin, HMB45, HPV (total) and HPV16. Ki67 labeling index was approximately 40%. High-throughput gene sequencing indicated a mutation in the C-kit gene. Additional IHC staining for DOG-1 and CD117 was positive (**Figures 3**), CD34 was also positive, CD31 and ERG was negative. The final diagnosis was gastrointestinal stromal tumor (epithelioid type) categorized as high-risk group.

**Figure 4:**
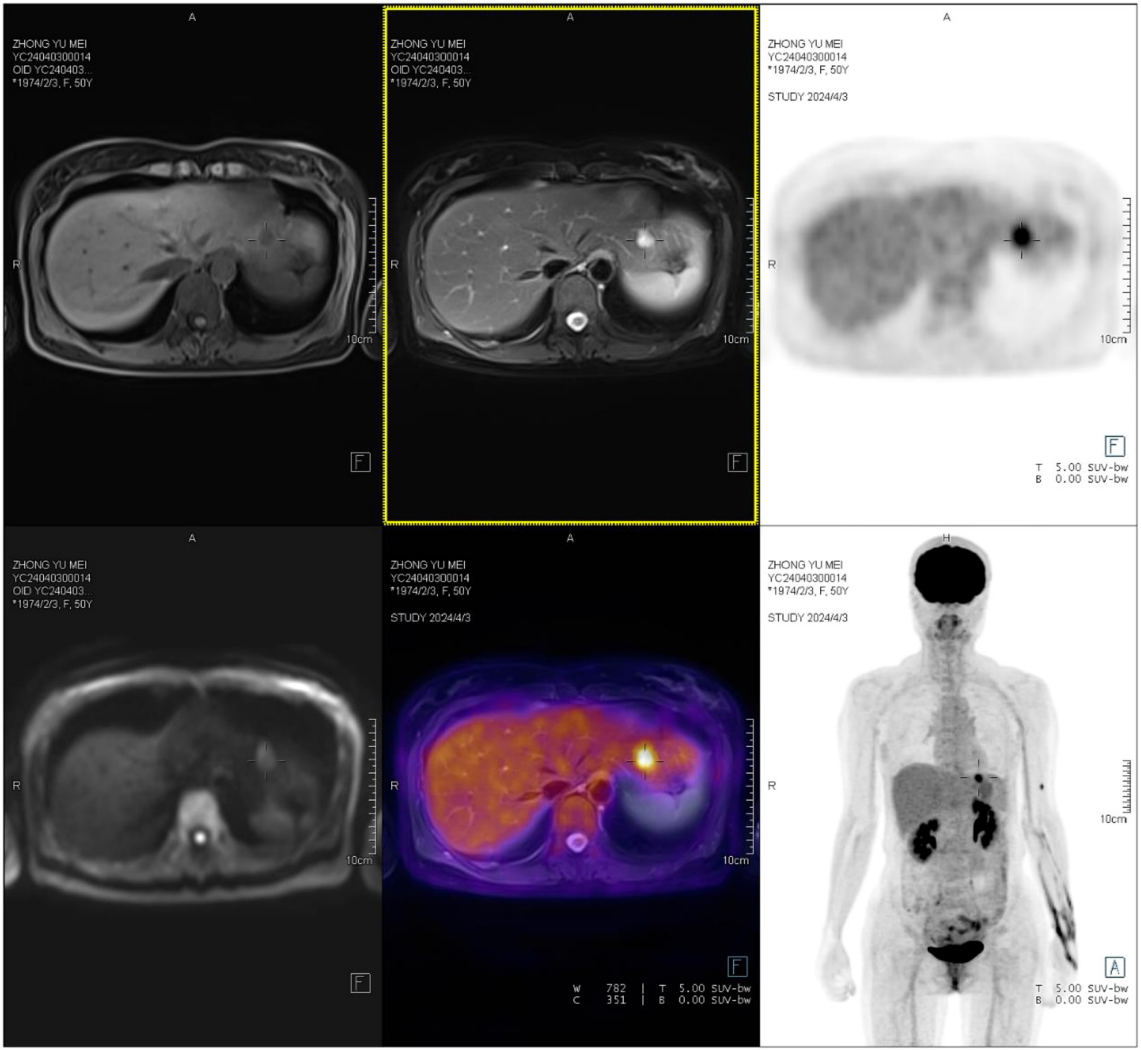
A low-density nodule is observed in liver segment II, with slightly low T1WI signals and slightly high T2WI signals. The nodule has clear boundaries and measures approximately 1.2×1.1 cm, with increased FDG uptake and an SUVmax of 8.4.

One month post-surgery, an enhanced CT scan of the entire abdomen revealed a slightly hypodense nodule in the left outer lobe of the liver, approximately 1.1 cm in diameter, with suspected nodular enhancement at the edges. No other significant abnormalities were detected. A follow-up gastroscopy showed no obvious abnormalities. Colonoscopy revealed a depressed lesion about 1.2 cm in size located approximately 3 cm from the anus in the rectum, with nodular elevation in the center, converging folds around the lesion, irregular vasculature, and friable tissue on biopsy. Pathological results indicated moderate chronic inflammation with focal erosion and granulation tissue hyperplasia. Further PET/MR/CT fusion imaging revealed a low-density nodule in liver segment II. The nodule had slightly low signal intensity on T1WI and slightly high signal intensity on T2WI, with clear boundaries and a size of approximately 1.2 x 1.1 cm. The lesion showed increased FDG uptake with an SUVmax of 8.4, suggesting liver metastasis (**Figure 4**). Subsequently, the patient underwent laparoscopic local resection of the left liver. Intraoperatively, a tumor in liver segment II, approximately 1.8 x 1.6 cm, was observed protruding from the liver surface. The cut surface was grayish-white, hard, homogeneous, and without a capsule. No lymphadenopathy was noted in the abdominal cavity, and other intra-abdominal organs appeared normal. Pathological examination of the left liver confirmed metastatic gastrointestinal stromal tumor (epithelioid type). The patient was then prescribed oral imatinib (the selective tyrosine kinase receptor inhibitor) at 400 mg per day. After 6-month follow-up, there were no signs of local recurrence based on physical examination and imaging studies. Imaging showed no progression of hepatic lesions, and the patient reported no specific discomfort.

## Discussion

Rectal GISTs are relatively rare, leading to a general lack of awareness among clinicians, particularly gynecologists. The clinical manifestations of rectal GISTs depend on the tumor’s location and size and are typically non-specific. These tumors usually originate from the submucosa or muscularis propria and exhibit extraluminal growth, rarely causing bowel obstruction. Due to the loose surrounding tissue and the rich vascular and neural network below the peritoneal reflection, early detection of these tumors is difficult, and they are often discovered only when they have grown significantly. When GISTs are located in the rectovaginal septum, they may be palpable through the vaginal wall. Imaging studies often lack specificity, and misdiagnosis as prostate cancer, vaginal malignancies, ovarian masses, or rectal cancer is common. The ultrasound typically reveals well-defined masses with clear boundaries and intact capsules, showing rich internal blood flow on Color Doppler Flow Imaging (CDFI). On unenhanced CT scans, the solid components of rectal GISTs appear isodense with muscle tissue, while MRI shows isointense signals on T1-weighted images and hyperintense signals on T2-weighted images. Enhanced CT or MRI scans typically show mild to moderate enhancement, and larger tumors often display heterogeneous enhancement due to internal hemorrhage or necrosis. Diagnosis relies on pathological examination and genetic testing. The genetic mutation profile of rectal GISTs is similar to that of GISTs in other locations. Genetic testing should at least include exon 9, 11, 13, and 17 of the c-kit gene and exon 12 and 18 of the PDGFRA gene. For patients with secondary resistance, testing should also include exon 14 and 18 of the c-kit gene. The expression of CD117 and DOG-1 is diagnostically significant, and it is recommended to use a combination of markers, including CD117, DOG-1, CD34, Succinate Dehydrogenase B (SDHB), and Ki67 (2). According to the NCCN guidelines, all GIST patients should undergo evaluation by a multidisciplinary team (MDT) with expertise in sarcoma diagnosis and treatment. Implementing MDT approach can facilitate optimal initial management and enhance patient outcomes.

The European Society for Medical Oncology (ESMO) Clinical Practice Guidelines state that the standard treatment for localized GIST is complete surgical resection (3). It is crucial to avoid rupturing the tumor’s pseudocapsule, ensuring the integrity of the resected tissue and achieving negative margins both macroscopically and microscopically. Perioperative imatinib and R0 resection are associated with improved recurrence-free survival. Conversely, R1 resection is linked to poorer recurrence-free survival, regardless of perioperative imatinib use (4). However, recent evidence suggests that positive microscopic margins are not an independent predictive factor for RFS in GIST. R1 resection may be considered a reasonable alternative to avoid major functional sequelae and should not lead to reoperation (5). According to the ESMO guidelines, if R1 excision was already carried out, a re-excision is not recommended on a routine basis (3). As GISTs rarely metastasize to lymph nodes, regional lymphadenectomy is unnecessary. Surgical approaches include local resection (LR) and radical excision (RE). Historically, rectal GISTs have often been treated with RE, resulting in significant trauma and severe bowel dysfunction. Recent studies have shown that LR and RE have equivalent long-term efficacy in treating rectal GISTs. However, even low-risk patients face a high recurrence rate, primarily manifesting as local recurrence (6, 7). LR approaches include transanal, perineal, abdominal, and sacrococcygeal routes, tailored to the individual patient’s condition. Female patients with GISTs located on the anterior rectal wall may undergo vaginal resection (8), although this approach is primarily documented in case reports. This could be due to preoperative misidentification of the tumor’s origin as being closely related to the reproductive tract (9) or being of uncertain origin (10). In other cases, rectal GISTs are confirmed, and vaginal resection is considered feasible following evaluation (11, 12). The vaginal wall, being a flexible and elastic tissue, provides a better surgical field. However, it is crucial to preserve the rectal mucosa as much as possible to prevent contamination of the surgical area with bowel contents, thereby reducing the risk of infection and rectovaginal fistula (13). In the current case, the primary lesion was successfully resected through a vaginal approach. The surgical trauma was minimal, recovery was quick, and the treatment results were satisfactory to the patient. More cases need to be accumulated to determine the indications for vaginal approach surgery for rectal GISTs.

Most scholars believe that GISTs occurring outside the stomach possess higher malignant potential (14). The selective tyrosine kinase receptor inhibitor imatinib has been proven to improve recurrence-free survival in patients with rectal GISTs (8). Additionally, neoadjuvant treatment with imatinib significantly reduces tumor size and mitotic index in rectal GISTs. Therefore, preoperative therapy is recommended for rectal GISTs that are expected to be incompletely resectable, require multi-organ resection, or cannot preserve the anus (15, 16). Currently, the NIH 2008 modified criteria are widely used for risk stratification of GISTs. Patients with certain nongastric tumors (2.1–5 cm and > 5 mitoses per 50 high-power fields or 5.1–10 cm and < or = 5 per 50 high-power fields) and those with tumor rupture are considered high risk, with a recurrence risk of more than 15% to 20% (17). Patients with very low or low-risk GIST can be closely monitored at the primary site following complete surgical resection. However, for those with intermediate or high-risk GIST, neoadjuvant or adjuvant molecular targeted therapy is recommended to facilitate successful surgery and prevent postoperative recurrence or metastasis. Before undergoing adjuvant therapy, genetic testing is recommended to identify specific mutations and guide the treatment plan. Imaging studies are crucial for evaluating the feasibility of complete resection. For most GISTs that can be completely resected without significantly affecting the function of adjacent organs, routine preoperative biopsy is not recommended.

Reports indicate that approximately 17% of GIST patients present with liver metastases at their initial diagnosis, and over 70% will develop liver metastases even after radical resection (18). However, compared to GISTs in other locations, rectal GISTs are more likely to experience local recurrence rather than distant metastases, such as liver metastases (6). Nevertheless, the primary cause of death following rectal GIST resection is not local recurrence but distant metastases, often involving the liver. Surgical resection of liver metastases of GISTs generally yields poor outcomes (19). In this case, liver metastasis was detected just one month post-surgery, highlighting the unpredictable aggressiveness of high-risk rectal GISTs and the need for cautious treatment and follow-up. Once diagnosed with advanced GIST (metastatic, unresectable, or recurrent), patients should immediately begin treatment with imatinib at a standard dose of 400 mg/day, regardless of the presence of symptoms. If disease progression occurs or in cases of GIST with KIT exon 9 mutations, the initial treatment dose can be increased to 800 mg/day (3, 20). Other approved treatments for GIST include sunitinib (Sutent) and ponatinib (14). Patients with PDGFRA exon 18 mutations must receive avapritinib as the first-line treatment (21). The optimal follow-up strategy remains inconclusive. According to the ESMO guidelines, high-risk patients can undergo a routine follow-up with an abdominal CT scan or MRI every 3–6 months for 3 years during adjuvant therapy, unless contraindicated, then on cessation of adjuvant therapy every 3 months for 2 years, then every 6 months until 5 years from stopping adjuvant therapy and annually for an additional 5 years. For low-risk tumors, a routine follow-up may be carried out with abdominal CT scan or MRI, for example, every 6–12 months for 5 years (3).

In summarizing this case, the main reasons for misdiagnosis were as follows: (1) The rectal stromal tumor was located in the rectovaginal septum and pressed against the vaginal wall, obscuring the boundary between the tumor and the vagina. This led to an incorrect determination of the primary organ in imaging studies. The complex and closely related structure of pelvic organs poses challenges and difficulties for the localization, characterization, and differential diagnosis of pelvic masses. Accurate localization is essential for correct characterization and precise evaluation. To determine whether a mass originates from the rectum, vaginal wall, or rectovaginal septum, ultrasound examination with transrectal or transvaginal probes can be used to observe whether there is a high-echo capsule between the mass and the rectum or vaginal wall. Additionally, using the probe to push the mass can help observe any relative motion between the mass and the rectum or vaginal wall. (2) The low incidence of rectal stromal tumors and insufficient awareness among gynecologists and radiologists are the primary reasons leading to misdiagnosis. Enhancing theoretical knowledge and summarizing clinical experience are effective methods to avoid misdiagnosis and mistreatment. Sometimes MDT approach is essential. If this patient had undergone a preoperative MDT discussion involving gynecology, colorectal surgery, and radiology, the preoperative preparation would have been more comprehensive.

Fortunately, after the diagnosis of rectal GIST, the patient received standardized treatment. Clinicians actively performed enhanced CT and PET/MR/CT scans, which revealed liver metastases, followed by surgical resection and subsequent imatinib therapy. Additionally, this patient’s surgical plan provides a reference for exploring the feasibility and indications of vaginal approaches for rectal GIST surgery. We will continue to follow up on this patient’s prognosis.

## Conclusions

Rectal gastrointestinal stromal tumors (GISTs) may be misdiagnosed as originating from other organs. MDT approach is essential for the diagnosis and management of GIST. Surgical resection remains the initial treatment for localized rectal GISTs, with local excision and radical resection showing similar prognoses. Female patients with GISTs located on the anterior rectal wall may be suitable for a vaginal approach surgery; however, the indications need further study. Imatinib neoadjuvant chemotherapy may be administered preoperatively if necessary. Once a rectal GIST is diagnosed, it is crucial to monitor for distant metastases, such as liver metastases, making enhanced CT or even PET-CT scans essential. Imatinib is the first-line treatment for recurrent or metastatic GISTs. As rectal GISTs are clinically rare, healthcare professionals should improve their understanding of its diagnosis and treatment to reduce misdiagnosis and missed diagnosis.

## Data Availability

The original contributions presented in the study are included in the article/supplementary material. Further inquiries can be directed to the corresponding author.
